# Resveratrol inhibits TNF-α-induced inflammation to protect against renal ischemia/reperfusion injury in diabetic rats ^1^

**DOI:** 10.1590/s0102-865020200050000006

**Published:** 2020-07-03

**Authors:** Min Wang, Xiaodong Weng, Hui Chen, Zhiyuan Chen, Xiuheng Liu

**Affiliations:** I PhD, Department of Urology, Renmin Hospital of Wuhan University, Wuhan, China. Conception and design of the study, acquisition and interpretation of data, manuscript writing.; II PhD, Department of Urology, Renmin Hospital of Wuhan University, Wuhan, China. Conception and design of the study; acquisition, analysis and interpretation of data; critical revision.; III PhD, Department of Urology, Renmin Hospital of Wuhan University, Wuhan, China. Acquisition of data, technical procedures, histopathological examinations.; IV PhD, Department of Urology, Renmin Hospital of Wuhan University, Wuhan, China. Acquisition of data, statistics analysis, manuscript preparation.; V PhD, Full Professor, Department of Urology, Renmin Hospital of Wuhan University, Wuhan, China. Design and supervised all phases of the study, critical revision, final approval.

**Keywords:** Ischemia, Reperfusion, Kidney, Resveratrol, Tumor Necrosis Factor-alpha, Rats

## Abstract

**Purpose:**

To examine effects of resveratrol on renal ischemia/ reperfusion injury (I/R) in a streptozotocin (STZ)-induced diabetic rat model.

**Methods:**

Twenty-four male Sprague Dawley rats were treated with STZ injection for the development of diabetes, and divided into the following groups: Sham group, I/R group and Resveratrol group (n=8). Resveratrol (RSV) was administered at a dose of 10 mg.kg-1.d-1 fourteen days prior to suffering from I/R. Renal function, histology, SOD, MDA, TUNEL assay and expression of TNF-α, IL-1β, NF-κB-P65, COX-2 and Caspase3, Bcl2 and Bax were analyzed.

**Results:**

Administration of RSV significantly reduced the serum levels of renal dysfunction and injury markers, including creatinine, blood urea nitrogen and MDA; in the other hand, it significantly increased the serum levels of SOD. The protective effect of RSV was also reflected on histologic evaluation. RSV reduced the number of apoptotic cells as determined by TUNEL assay. RSV significantly reduced the protein expression of TNF-α, IL-1β, NF-κB-P65, COX-2 and Caspase3, and Bax. Meanwhile, RSV significantly increased the protein expression of Bcl2.

**Conclusion:**

RSV attenuated I/R-induced renal injury in diabetic rats through the modulation of oxidative stress and TNF-α-stimulated inflammation.

## Introduction

Acute kidney injury (AKI) is encountered in many clinical situations and leads to increase of health costs. As we know, renal ischemia/reperfusion (I/R) is one of the major causes of AKI. Diabetes is a common and increasing chronic metabolic disease. Diabetic nephropathy might gradually and unavoidably develop to end-stage renal failure^1^ . Diabetes has been identified as an independent risk factor for AKI^2^ ; meanwhile, it might not only cause renal damage and dysfunction, but also exacerbated oxidative stress, inflammation and apoptosis of I/R^3 - 5^ .

The development mechanism of renal I/R is very complicated. Firstly, it is characterized by energy deficit, and then, it is followed by a series of physiological and pathological changes, including oxidative injury, inflammation, and metabolic dysfunction during reperfusion^6^ . The underlying mechanism of I/R includes reactive oxygen species (ROS), abnormal lipid metabolism, inflammatory cascade, calcium overload and nitrosoredox imbalance from ischemic tissues. The tumor necrosis factor-α (TNF-α) was a major pro-inflammatory cytokine in kidney pathogenesis. Some studies have shown that TNF-α plays a key role in inflammation after renal I/R by up-regulating inflammatory genes such as cyclooxygenase 2 (COX-2)^7^ . COX-2 plays an important role in the development of a serious of renal inflammatory diseases, especially in renal I/R. Inhibiting TNF-α activity has proved to have antioxidant and anti-inflammatory effect and protects kidneys from ischemic injury^8^ .

Resveratrol was a polyphenol, which has been reported to possess a kind of pharmacological effects, such as anti-inflammatory properties, protection against coronary heart disease, modulation of lipid metabolism and prevention of cancer^9 - 11^ . Resveratrol has significant protective effects against I/R injury in various organs^12 - 16^ . Specifically, resveratrol has shown to protect against several types of renal injury by inhibiting oxidative stress and ameliorating inflammatory response, including diabetic nephropathy, drug-induced injury, and I/R injury^17 - 18^ .

Nowadays, there is no research that reports the effects of resveratrol on renal ischemia/reperfusion injury in diabetic rats. In the present study, we aimed to investigate the effects of administration of resveratrol before I/R on renal structural and functional changes, oxidative stress, inflammatory response and apoptosis in diabetic rats.

## Methods

### Animal preparation

Adult male Sprague-Dawley rats (180-250g) were obtained from the experimental animal center of Union Hospital Affiliated to Tongji Medical College, Huazhong University of Science and Technology. The committee for experimental animals of Wuhan University approved all experimental procedures, and the procedures complied with the Guidelines for the Care and Use of Laboratory Animals.

Experimental diabetes mellitus was induced by a single intraperitoneal injection of STZ at the dose of 50 mg/kg. It was prepared in 0.1 mol/l citrate buffer (pH 4.5), immediately before use. The blood samples at the 24th hour after STZ injection were obtained from the caudal veins. Rats with a glucose concentration higher than 300 mg/dL were considered as diabetic. Successfully, there was no rat death when the diabetes model was made. Then, these diabetic rats were randomly divided into three groups (8 rats per group, n = 8): Sham group, I/R group and Resveratrol group. In the Sham group, the kidneys were treated identically with I/R group, without clamping the left pedicle; in the ischemic/reperfusion injury (I/R) group, the midline laparotomy was performed and, after right nephrectomy, the left kidney was subjected to 45 minutes of ischemia followed by reperfusion; in the Resveratrol group, resveratrol (RSV) was administered at a dose of 10 mg.kg^- 1^ .d^- 1^ orally fifteen days prior to suffering I/R. In brief, the operation was performed under fully maintained anesthesia with pentobarbital (45 mg/kg).

These diabetic rats were killed 24 hours after reperfusion. Blood was obtained via puncture of the inferior vena cava, and the left kidney was removed under fully maintained anesthesia. After removal, the kidneys were fixed in 10% phosphate-buffered formalin or immediately frozen, and stored at -80°C for different procedures.

### Renal function analysis

Serum creatinine (Cr) and blood urea nitrogen (BUN) were determined, using standard techniques and an Olympus AU2700 Analyzer (Olympus, Optical Co., Tokyo, Japan).

### Histologic examinations

For histologic preparations, kidneys were fixed in 4% paraformaldehyde, paraffin embedded, and sectioned into 5µm thick sections according to the standard procedure. The sections were deparaffinized and hydrated gradually, and stained with hematoxylin and eosin (H&E). Morphologic assessments were performed blindly by an experienced renal pathologist.

### Apoptosis assay

Renal apoptosis was examined by TUNEL assay using the in situ Apoptosis Detection kit from Roche Applied Science. TUNEL-positive cells were identified through the nucleus, which was stained either tan or brown. Five fields were randomly selected and the apoptosis index was calculated as the ratio of apoptotic-to-total cells.

### Measurement of oxidative stress

The malondialdehyde (MDA) levels and superoxide dismutase (SOD) were detected using commercially available kits (Jiancheng Biotech, Nanjing, China), according to the manufacturer’s instructions.

### Immunohistochemistry

The expressions of COX-2 and Caspase3 were investigated. Tissue sections were stained by immunohistochemistry (IHC) using specific antibodies for COX-2 (1:1000 dilution; Cell Signaling Technology, Boston, MA) and Caspase3 (1:1000 dilution; Cell Signaling Technology, Boston, MA). Serial sections (thickness 5µm) were cut from the tissue blocks, deparaffinized in xylene, and hydrated in a graded series of alcohol. Staining was then performed using the DAB chromogenic agent (Dako Corp, Carpinteria, CA). Negative control experiments were routinely performed. For quantitation, the relative mean integrated optical density (IOD) of each group was divided by the average IOD of the control. All slides were evaluated by an experienced renal pathologist who was unaware of the origin of the slides.

### Western Blot analysis

The protein expression levels of TNF-α, IL-1β, NF-κB-P65, COX-2 and Caspase3, Bcl2 and Bax were examined by Western blotting. Briefly, proteins were extracted from kidneys, separated on 10-12% SDS-PAGE gels (40 ug/lane) and then transferred to nitrocellulose membrane (Bio-Rad, Hercules, CA). The membranes were blocked with 5% nonfat milk in TBST buffer (10 mmol/L Tris-HCl, 0.15 mol/L NaCl, and 0.05% Tween 20, pH 7.2) for 2h and incubated with primary antibodies overnight at 4°C. Primary antibodies used here were monoclonal mouse antibodies against TNF-α (1:1000 dilution; Santa Cruz Biotechnology), IL-1β (1:1000 dilution; Santa Cruz Biotechnology, Santa Cruz, CA), NF-κB-P65 (rabbit monoclonal, 1: 100, Cell signal technology), and COX-2 (1:1000 dilution; Cell Signaling Technology, Boston, MA), Caspase3 (1:1000 dilution; Cell Signaling Technology, Boston, MA), Bcl2 (1:1000 dilution; Cell Signaling Technology, Boston, MA) and Bax (1:1000 dilution; Cell Signaling Technology, Boston, MA). After extensive washing with TBST buffer, the membranes were incubated with HRP-conjugated anti-mouse or anti-rabbit secondary antibodies (1:2000 dilution; Santa Cruz Biotechnology). The proteins were detected using an enhanced chemiluminescence system (ECL kit, Pierce Biotechnology, Beijing, China) and captured on light-sensitive X-ray film (Kodak, Shanghai, China). Optical densities were detected using ImageJ software.

### Statistical analysis

All data are presented as mean±SEM. The means of the different groups are compared using one-way analysis of variance (ANOVA) and the Student-Newman-Keuls test. The Kruskal-Wallis ANOVA on ranks is used for nonnormally distributed data. The level of statistically significance is set at P<0.05.

## Results

### Renal function

**Figure 1:**
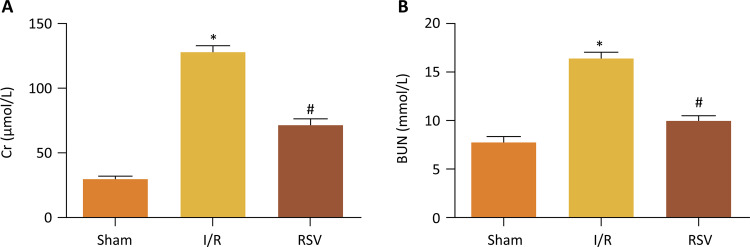
- Cr and BUN levels after treatment of 24h. Cr and BUN levels of I/R group and RSV group are both higher than that of Sham group, but RSV group significantly reduced Cr and BUN levels compared to I/R group.(*P<0.05 *vs.* Sham group, #P<0.05 *vs.* I/R group).

Cr and BUN levels were measured at 24 hours following I/R in diabetic rats. In this model, renal I/R caused a marked increase in serum Cr as well as BUN after I/R in diabetic rats. Treatment with resveratrol significantly reduced the serum Cr and BUN levels ( Fig. 1 ).

### Morphologic features and immunohistochemistry

**Figure 2:**
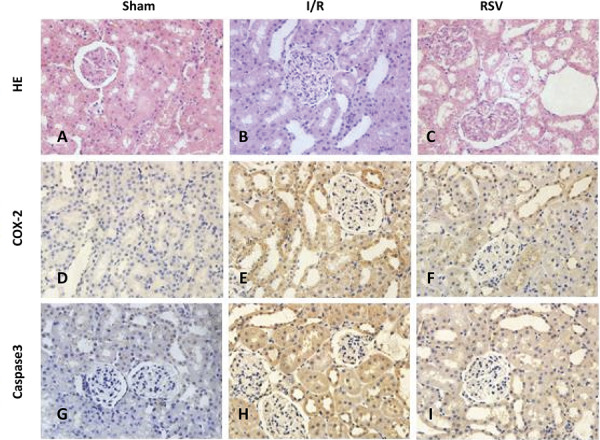
- Histological evaluations and immunohistochemical expression of COX-2 and Caspase3 in renal tissues (magnification, 400). (A) H&E staining from Sham group rats. (B) H&E staining from I/R group rats. (C) H&E staining from RSV group rats. (D) Expression of COX-2 in Sham group rats. (E) Expression of COX-2 in I/R group rats. (F) Expression of COX-2 in RSV group rats. (G) Expression of Caspase3 in Sham group rats. (H) Expression of Caspase3 in I/R group rats. (I) Expression of Caspase3 in RSV group rats.

Morphologic features were evaluated using H&E ( Fig. 2 ). The Sham group did not show any morphological changes. By contrast, the kidneys of untreated ischemic diabetic rats showed tubular cell swelling, cellular vacuolization, pyknotic nuclei, medullary congestion, and moderate to severe necrosis. Treatment with resveratrol preserved the normal morphology of the kidney and showed slight edema of the tubular cells and mild necrosis.

In our study, COX-2 and Caspase3 were detected by immunohistochemistry staining ( Fig. 2 ). It revealed that COX-2 (IOD 1.00±0.03) and Caspase3 (IOD 1.01±0.02) were rarely found in Sham group. But in I/R group, renal tissues were strongly positive for COX-2 (IOD 4.49±0.16) and Caspase3 (IOD 8.39±0.23) expressions. Compared with the I/R group, COX-2 (IOD 2.31±0.13) and Caspase3 (IOD 4.46±0.20) expressions were ameliorated by resveratrol in RSV group (P < 0.01) ( Fig. 2 ).

### Measurement of MDA and SOD

**Figure 3:**
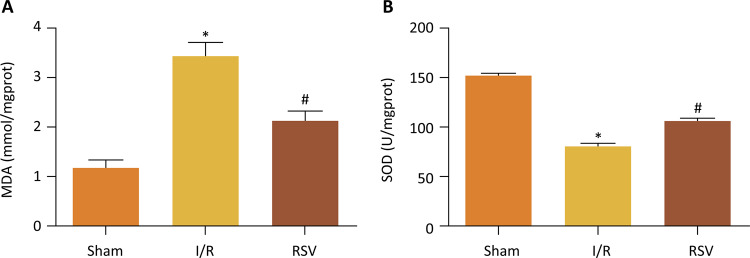
- MDA and SOD levels at 24h after reperfusion. MDA levels of I/R group and RSV group are both higher than that of Sham group, but RSV group significantly reduced MDA levels compared to I/R group. SOD levels of I/R group and RSV group are both lower than that of Sham group, but RSV group significantly increased SOD levels compared to I/R group. (*P<0.05 *vs.* Sham group, #P<0.05 *vs.* I/R group)

Renal I/R significantly increased the enzymatic activity of MDA, but it significantly decreased the enzymatic activity of SOD. However, these changes were significantly reversed by treatment with resveratrol ( Fig. 3 ).

### Resveratrol decreased TNF-a and Cox2 expression

**Figure 4:**
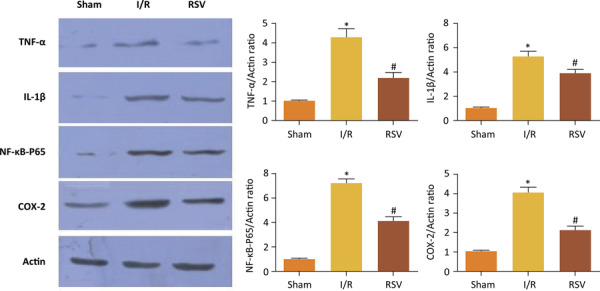
Change in expression TNF-α, IL-1β, NF-κB-P65 and COX-2 protein determined by Western blot analysis in Sham group, I/R group and RSV group. (*P<0.05 *vs.* Sham group, #P<0.05 *vs.* I/R group)

To investigate the expression of TNF-α, IL-1β, NF-κB-P65 and COX-2 in diabetic rats, we measured the levels of TNF-α, IL-1β, NF-κB-P65 and COX-2 by Western Blot ( Fig. 4 ). The expression of TNF-α, IL-1β, NF-κB-P65 and COX-2 were significantly greater in I/R group than in Sham group. However, resveratrol treatment inhibited the expression of TNF-α, IL-1β, NF-κB-P65 and COX-2 after renal I/R in diabetic rats.

### Resveratrol reduced cell apoptosis after renal ischemia/reperfusion

**Figure 5:**
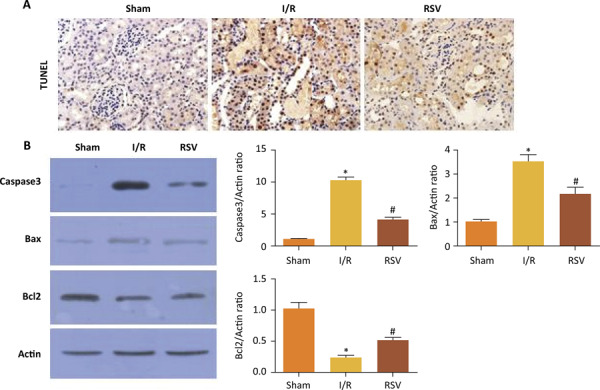
(A) Representative TUNEL assay obtained 24 hours after reperfusion (magnification, 400). (B) Change in expression Caspase3, Bax and Bcl2 protein determined by Western blot analysis in Sham group, I/R group and RSV group. (*P<0.05 *vs.* Sham group, #P<0.05 *vs.* I/R group)

Apoptosis was evaluated by TUNEL assay. At 24 hours after I/R, a small quantity of TUNEL-positive cell was present in kidney obtained in Sham group. TUNEL assay showed an increase in the number of apoptotic cells after I/R and a decrease from RSV group ( Fig. 5 ).

Renal I/R injury significantly up-regulated the levels of active caspase-3 and Bax expression in kidneys of diabetic rats. Resveratrol treatment inhibited the expression of active caspase-3 and Bax in diabetic rats. Meanwhile, Renal I/R injury significantly reduced cytosolic Bcl-2 levels in kidneys of diabetic rats. Resveratrol treatment restored the levels of Bcl-2 in diabetic rats ( Fig. 5 ).

## Discussion

Although resveratrol had demonstrated to possess significant protective effects against I/R injury of various organs, including kidney^12 - 18^ , we demonstrated for the first time the protective effects of resveratrol in diabetic rats after renal I/R injury. Our study showed that administration of resveratrol proved to be an effective treatment against renal I/R in diabetic rats.

We demonstrated that resveratrol significantly reduced serum markers of renal dysfunction and injury, such as Cr and BUN. This was further supported by the preservation of renal histologic architecture in the treatment groups when compared to the Sham group and I/R group. Meanwhile, treatment with resveratrol was associated with a significant reduction in pro-inflammatory cytokines TNF-α, IL-1β and COX-2. Furthermore, administration of resveratrol was associated with a significant reduction in apoptosis induced by I/R injury in diabetic rats.

Oxidative stress played a key role in renal I/R injury^19 , 20^ . As we know, free radicals played an important role in the process of lipid peroxidation reactions; it is very effective to protect renal function by inhibiting the oxidative stress-induced peroxidation of membrane lipids and other target macromolecules. During the reperfusion phase of ischemia, several potential sources of toxic oxygen species were involved in the injury tissue. ROS was one of these toxic oxygen species. It could not only deactivate some antiproteases, but also activate some proteases, which could lead to tissue damage^21 , 22^ . Hyperglycemia was a notable characteristic of diabetes and could elevate the basal level of ROS in tissues and promote chronic oxidative stress. In the present study, MDA, an index of lipid peroxidation, was increased, but SOD was decreased in the kidney tissues, indicating the presence of I/R-induced oxidative damage in diabetic rats. In accordance with the previous observations, the findings of the present study showed that resveratrol was effective to reduce the I/R-induced renal damage and dysfunction in diabetic rats by reducing lipid peroxidation and scavenging oxygen free radicals.

Inflammatory response also played an important role in renal I/R injury. In the process of I/R injury, massive damage associated molecules were released. Then, inflammatory cells were recruited from the circulation into the injury renal tissue^23^ . This was usually accompanied by the release of inflammatory cytokines, such as TNF-α, IL-1β and IL-6. Studies^4 , 24^ showed that the inflammatory response induced by I/R injury was further exacerbated in diabetic rats, in which TNF-a and IL-1β levels in serum or renal tissues increased significantly. In this study, we have demonstrated that administration of resveratrol proved to be effective to inhibit the expression of the pro-inflammatory cytokines TNF-α and IL-1β elevated in the kidney of diabetic rats after I/R injury. Furthermore, COX-2 played an important role in renal inflammation, which was regulated by various stimuli, including TNF-α. Down-regulation of COX-2 proved to alleviate renal inflammation and reduce renal damage^25^ . In the present study, resveratrol treatment proved to inhibit the expression of TNF-α, IL-1β, NF-κB-P65 and COX-2 after renal I/R in diabetic rats. So, we speculate that resveratrol might attenuate inflammatory response after renal I/R injury in diabetic rats via inhibiting TNF-α-induced NF-κB signaling and COX-2 activation.

Renal apoptosis was an important prognosticator in the development of ARF induced by I/R injury^26^ . As oxidase load increased in the mitochondria, the outer membrane of mitochondria tended to be more permeable, resulting in the translocation of Bax from cytosol to the mitochondri. The Bcl-2 family proteins took part in the process of pro-apoptotic proteins translocation^27^ . Our results showed that resveratrol significantly inhibited apoptosis caused by renal I/R injury in diabetic rats. In order to further clarify the reason of this change, we investigated the expressions of key apoptotic-related molecules, including active caspase-3, Bax and Bcl2. Our study showed that resveratrol increased the expression of anti-apoptotic Bcl-2 protein and inhibited the levels of Bax and active caspase-3.

## Conclusions

We demonstrated that resveratrol protected diabetic rats against I/R-mediated renal injury. We further demonstrated that resveratrol possessed anti-oxidant, anti-inflammatory and anti-apoptotic properties after renal I/R injury in diabetic rats, which might be mediated via the modulation of oxidative stress, inhibition of TNF-α-stimulated inflammation and inactivation of COX-2. These findings suggest the potential role of resveratrol against renal injury in diabetic patients.

## Footnotes

1 Research performed at Department of Urology, Renmin Hospital of Wuhan University, Wuhan, China.
